# Contactless monitoring for the elderly: potential and pitfalls

**DOI:** 10.1093/sleep/zsad227

**Published:** 2023-09-02

**Authors:** Ju Lynn Ong, Kelly Glazer Baron

**Affiliations:** Sleep and Cognition Laboratory, Centre for Sleep and Cognition, Yong Loo Lin School of Medicine, National University of Singapore, Singapore, Singapore; Department of Family and Preventive Medicine, University of Utah, Salt Lake City, UTUSA

The rapid rise of consumer sleep technologies is advancing the potential for large-scale, longitudinal sleep monitoring that could pave the way for the development of personalized recommendations, early identification of risk factors, and monitoring of disease progression [1, 2]. While clinical polysomnography has been traditionally conducted among individuals who are seeking evaluation for a sleep disorder, and over a short period (e.g. overnight at a sleep lab with polysomnography or 1-week at-home with actigraphy), these lower-cost, easy-to-use consumer devices are now readily accessible to all, enabling longitudinal measurements to be performed within the comfort of one’s own home.

Contactless technologies (CTs) or “nearables” have been in the market for more than a decade, and unlike their “wearable” counterpart that can be obtrusive and require periodic charging/synching to a smartphone app, CTs are an attractive alternative to those who find wearables uncomfortable, and the automatic recording may be helpful for less tech-savvy users and those with limited mobility or ability to synch a device, e.g. in patients with severe medical or psychiatric disorders, and older adults in nursing homes [3–5]. Devices can also be plugged in continuously with data streamed to a cloud, requiring little effort from users to charge and synch the devices. While two main forms have been proposed since its inception, notably (1) bedside devices using camera [6], radar [7], sonar [8], or thermography-based technologies [9], and (2) under-mattress devices based on piezoelectric or pressure sensors [10, 11], the latter is less susceptible to interference from other electromagnetic sources or peripheral objects in the bedroom and is the preferred method for users concerned with privacy in the bedroom setting [12]. In addition, while all CTs are designed to track sleep and wake transitions [13, 14], a handful can also monitor sleep stages due to its added heart-rate tracking capabilities over conventional actigraphy [7, 11, 13, 15].

In this issue of *SLEEP*, Ravindran et al. [16] add on to the existing body of CT evaluation studies by comparing the performance of two different CTs (the EmFit QS and the Withings Sleep Analyzer) in a free-living setting over 7–10 days, to the current standard of longitudinal sleep assessment at-home, i.e. actigraphy combined with sleep diaries. Although a previous study evaluated the performance of the EmFit QS against polysomnography [15], this was done on a single night under laboratory conditions, and in sleep-disordered patients. Performance was poor, with significant overestimation of total sleep time (TST; 177.5 minutes) and underestimation of wake after sleep onset (WASO; 44.74 minutes) but sleep onset latencies (SOL) and average resting HR estimation were comparable to the gold standard. The present sample was instead drawn from community-dwelling older adults in whom ease of monitoring is paramount, and where some trade-offs for accuracy could be acceptable. Sleep diaries are burdensome to complete, and can be subject to recall biases. In addition, there is merit in comparing subjective estimates to objective ones, as the degree of sleep misperception could itself be a reflection of sleep quality rather than just quantity, and be an important target to intervene upon [17].

All sleep periods, including nocturnal, and nap episodes were considered in the evaluation, as is important for the monitoring of longitudinal, 24-hour rest-activity-rhythms. The authors show that while bed presence (i.e. going into bed, getting out of bed, and time-in-bed [TIB]) estimates were comparable to sleep diary measures (ICCs > 0.75), nocturnal TST and SOL were significantly overestimated (TST: 92–101 minutes; SOL: 8–13 minutes) while WASO was underestimated (4–44 minutes) compared to sleep diary measures, leading to a ~9% overall overestimation of sleep efficiency estimates. Actigraphy still outperformed both devices for TST, SOL, and sleep efficiency estimation, and was better than Withings for WASO but worse than the EmFit QS. All three devices also estimated overall 24-hour TST duration with an error of ~10% compared to the sleep diary. However, the Emfit device missed detection of certain nap periods; only 11/29 in-bed naps were detected.

While comparisons to PSG should also be carried out in a similar way to Kholgi and colleagues [15] one could expect trends for TST and WASO to be quite similar given the overall poor sleep–wake classification of CTs and tendency toward poorer and more fragmented sleep in older adults.

What then can we conclude about the utility of CTs to date? Compared to their wearable counterparts that are in direct contact with the body, CTs still fall short in terms of accurate sleep–wake classification. At present, it would seem that it is mainly accurate for the 24-hour monitoring of when someone is in bed versus not (i.e. bedtime/wake time/TIB metrics), and not for TST or WASO estimation, which would be critical for monitoring the evolution of sleep disorders across time. In individuals who engage in non-sleep-related activity in bed, e.g. reading, texting, or watching television [18], solely relying on TIB tracking might also not give an accurate assessment of sleep–wake patterns. The presence of bed partners could also further complicate CT measurement [19]. Naps that were not taken in bed, were also not able to be detected by CTs, highlighting a significant shortcoming of the device, especially as daytime napping is more prevalent in older adults than it is in other age groups [20]. Nonetheless, across days, these metrics could still give a picture of regularity of in-bed/rise times, or any evidence of circadian phase shifts over time that could warrant an intervention.

In addition, although not discussed in the present work, tracking of cardiovascular metrics and movement patterns during sleep could also pave the way for integrated arrhythmia detection and other cardiovascular events [21–23], influenza/coronavirus disease monitoring [24], as well as, fall detection [25] in these vulnerable groups that could be shared in real-time with a caregiver or clinical provider. As aging in place becomes more common, one needs to carefully evaluate cost–benefit trade-offs associated with a particular use case of CTs. If its intended purpose is to provide real-time feedback in at-risk populations, e.g. truck drivers in a fatigue management program, then CTs may not yet be ready for deployment. However, given increasing socioeconomic and healthcare costs in older populations, a simple, low-cost, and easy-to-use digital tool could allow for the epidemiologic evaluation of sleep metrics and early detection of health deterioration or disease progression (e.g. Alzheimer’s disease) that is linked to poor sleep outcomes [1, 26].

Finally, even as the performance evaluation of CT is showing improvement over time in the ability to estimate sleep–wake patterns, it is important to consider the impact of automatic tracking versus more intentional methods such as synching a wearable or using paper and pencil or digital sleep diaries. If the CT data are to be used for behavior change interventions such as cognitive behavioral therapy for insomnia (CBT-I), CT users will need to be engaged with their data in a way that promotes feedback and adherence needed for sleep improvement.

In summary, the use of multi-day, naturalistic assessments in an older adult sample is an important next step in the potential for CT in sleep research. Having a thoughtful dialogue about the potential advantages and disadvantages as well as sources of bias are critical to advancing the possibilities of large-scale data collection in sleep research.
